# Artificial Intelligence Decodes Brain Elemental Signatures to Stratify Aging and Neurological Diseases

**DOI:** 10.34133/research.1283

**Published:** 2026-05-13

**Authors:** Augustin Tillement, Eszter Nemeth, Laurent David, Dilek Cenesiz, Ulrich Neumayer, Mathias Fousse, Yang Liu, Walter J. Schulz-Schaeffer, Klaus Fassbender, Alexandre Detappe, François Lux, Sergiu Groppa, Olivier Tillement, Yann Decker

**Affiliations:** ^1^ Ingénierie des Matériaux Polymères (IMP), UMR 5223, Universite Claude Bernard Lyon 1, INSA de Lyon, Université Jean Monnet, CNRS, F-69622 Villeurbanne, France.; ^2^ Nano-H, F-69270 Fontaines-Saint-Martin, France.; ^3^ Institut Lumière Matière (ILM), UMR5306, Universite Claude Bernard Lyon 1, CNRS, F-69100 Villeurbanne, France.; ^4^ Department of Neurology, Saarland University Medical Center, D-66421 Homburg, Germany.; ^5^ Department of Neurology, SHG Klinikum Merzig, D-66663 Merzig, Germany.; ^6^ Institute of Neuropathology, Medical Faculty of the Saarland University, D-66421 Homburg, Germany.; ^7^ Nanomedicine Laboratory, Institut de Cancérologie Strasbourg Europe, F-67000 Strasbourg, France.; ^8^ Institut Universitaire de France (IUF), F-75005 Paris, France.

## Abstract

The elemental composition of brains changes progressively with age, yet these metallome alterations remain largely unexplored as diagnostic biomarkers in neurological disease. Here, we present a comprehensive analysis of 24 inorganic elements in paired cerebrospinal fluid and serum samples from 1,608 individuals spanning healthy aging through 14 neurological conditions, representing the largest systematically standardized cohort for neurological metallomics. Uniquely, our unselected, consecutively admitted clinical cohort captures the full heterogeneity of neurological presentations, overcoming the limitations of traditional case–control designs focused on isolated disease entities. Machine learning analysis reveals that aging is associated with distinct cerebrospinal fluid elemental signatures independent of peripheral blood changes, primarily reflecting blood–brain barrier permeability alterations that correlate with established albumin quotient measurements. We identify 2 predominant patterns of neurological elemental dysregulation: one mainly consistent with passive barrier-mediated leakage in inflammatory conditions, and another mainly indicative of disease-intrinsic perturbations of metal homeostasis in neurodegenerative disorders. Age-stratified analysis reveals that elemental signatures evolve differently across the lifespan for distinct pathological processes. The integration of elemental signatures with routine clinical parameters through ensemble learning approaches enhances diagnostic accuracy across all tested neurological categories, establishing metallomics as a complementary biomarker class that captures orthogonal pathophysiological information. These findings establish brain metallomics as an emerging field where artificial intelligence reveals complex multi-element interactions present in neurological aging, opening new avenues for precision medicine in age-related neurological disorders.

## Introduction

Neurological diseases represent the leading cause of disability worldwide, yet diagnostic precision remains constrained by reliance on clinical symptoms that emerge after irreversible pathological changes have occurred [1]. The heterogeneous nature of neurological conditions, ranging from acute insults like stroke to chronic progressive diseases such as Alzheimer’s disease, demands biomarkers that can capture disease-specific molecular signatures suitable for clinical implementation [2–6]. While cerebrospinal fluid (CSF) provides direct access to central nervous system (CNS) pathophysiology [7,8], systematic elemental profiling has been severely limited by small sample sizes and fragmented single-disease studies [9,10], despite the established roles of inorganic elements in neuronal signaling and oxidative stress homeostasis [11–13]. Population aging further compounds these challenges [14], as age-related changes in element homeostasis may confound disease-specific signatures, yet large-scale, normative CSF element data across the adult lifespan are lacking [15–17]. Here, we performed a comprehensive inductively coupled plasma mass spectrometry (ICP-MS) analysis of 24 inorganic elements in paired CSF and serum samples from 1,608 individuals spanning 14 neurological conditions and age-matched controls, representing the largest systematically standardized cohort for such analysis. Our unselected, consecutive admission design captures the full heterogeneity of real-world neurological presentations, transcending the limitations of traditional case–control designs focused on isolated disease entities. We reveal that CSF element composition undergoes systematic age-related changes independent of serum trends, correlating with blood–brain barrier (BBB) permeability alterations accompanying normal aging [16]. Employing age-matched controls as normative baselines, we identify distinct disease-associated element signatures that successfully stratify neurological conditions through both conserved dysregulation patterns and unique disease-related perturbations. Machine learning integration with routine clinical parameters demonstrates enhanced diagnostic precision, establishing elemental profiling as a cost-effective first-line screening platform that can guide targeted testing with specialized biomarkers, thereby optimizing diagnostic workflows in aging populations.

## Results and Discussion

### Age-related elemental changes in CSF correlate with BBB permeability

**Fig. 1.**
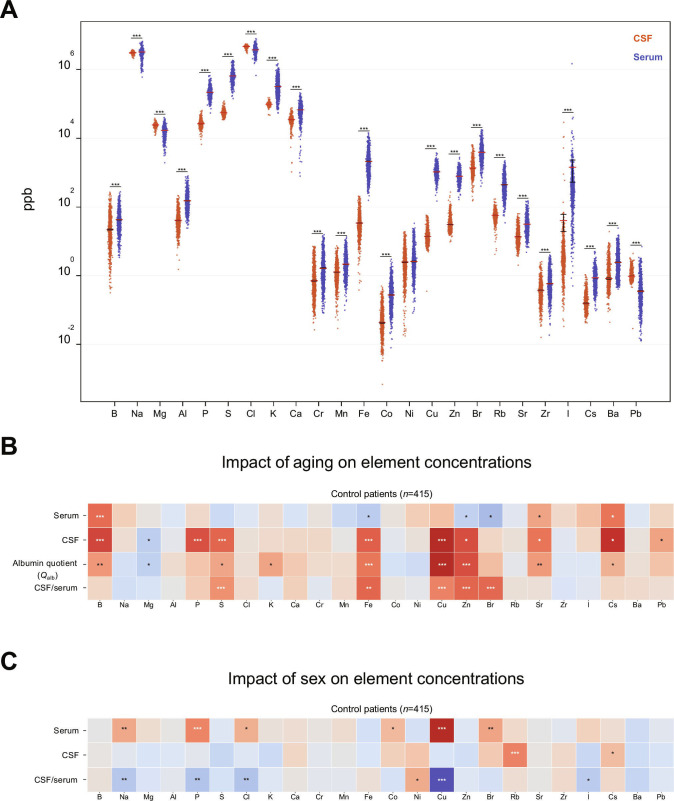
Demographic and compartment-specific elemental profiles in human biofluids. (A) Comparative concentrations (log_10_-transformed) of 24 elements measured in CSF (orange) and serum (blue) across all participants (*N* = 1,608). Individual data points are shown, with red horizontal bars indicating group means and flanking lines representing the standard error of the mean (SEM). (B and C) Associations between age (B) or sex (C) and element concentrations across biofluids in control individuals (*N* = 415), stratified by serum, CSF, albumin quotient, and CSF-to-serum ratios. (C) Color intensity represents direction and magnitude of association (red, positive association or higher in females; blue, negative association or higher in males). The albumin quotient row in (B) reflects associations between individual element concentrations and continuous albumin quotient values, not between elements and age, capturing co-variation with blood–CSF barrier permeability rather than age effects per se. Albumin quotient is included within this age-association panel because physiological aging is accompanied by a well-documented rise in blood–CSF barrier permeability. In (B) age-related alterations were most pronounced in the CSF, where Mg levels declined, while P, S, Fe, Cu, Zn, and Pb showed CSF-specific increases. In (C), sex-associated differences were strongest in serum, particularly for Na, P, Cl, Co, Cu, and Br, whereas only Rb and Cs showed CSF-specific sex effects. Statistical significance is indicated by asterisks (*P < 0.05, **P < 0.01, ***P < 0.001). A 2-sided Wilcoxon rank-sum test was used to compare element concentrations between CSF and serum (A) and between sexes (C); in (B), associations with age and albumin quotient were assessed by Spearman rank correlation.

To establish a comprehensive reference framework for neurological biomarker development, we quantified 24 inorganic elements using ICP-MS in paired serum and CSF samples from a total of 1,608 individuals. This cohort comprised 415 neurologically asymptomatic controls and 1,193 patients representing 14 distinct neurological conditions across the diagnostic spectrum. The comprehensive clinical and demographic profiles for the 1,608 individuals included in this study are presented in Table 1. Element concentrations exhibited a broad range, spanning 6 orders of magnitude while maintaining consistent abundance patterns between systemic and CNS compartments (Fig. 1A). Major elements such as the cations sodium (Na), magnesium (Mg), potassium (K), and calcium (Ca) along with the anions chlorine (Cl), phosphate (P), and sulfate (S) forms were predominant in both biofluids. In contrast, elements such as transition metals [chromium (Cr), manganese (Mn), iron (Fe), cobalt (Co), nickel (Ni), copper (Cu), zinc (Zn)], metalloids [boron (B)], post-transition metals [aluminum (Al), lead (Pb)], alkali and alkaline earth metals [rubidium (Rb), strontium (Sr), cesium (Cs), barium (Ba)], halogens [bromine (Br), iodine (I)], and other trace elements [e.g., zirconium (Zr)] were present at lower concentrations. Importantly, this hierarchical organization was preserved across compartments, indicating conserved physiological regulatory mechanisms governing element homeostasis.

**Table 1.** Demographic distribution across categories

Diagnosis	Total (*N*)	Age (mean ± SD)	Male *N*, (%)	Female *N*, (%)
Alzheimer’s disease	46	69.8 ± 11.2	25 (54.3)	21 (45.7)
Non-Alzheimer’s dementia	127	69.7 ± 10.7	72 (56.7)	55 (43.3)
Parkinson’s disease	80	73.3 ± 9.0	46 (57.5)	34 (42.5)
Other degenerative (SA, ALS)	32	60.3 ± 14.5	22 (68.8)	10 (31.2)
CNS infection	119	57.5 ± 19.1	88 (73.9)	31 (26.1)
Multiple sclerosis	25	45.6 ± 12.5	1 (4.0)	24 (96.0)
Autoimmune CNS inflammation	56	45.9 ± 16.1	29 (51.8)	27 (48.2)
Psychiatric disorders	81	45.2 ± 16.4	35 (43.2)	46 (56.8)
Other neurological (abnormal CSF)	89	58.9 ± 16.9	58 (65.2)	31 (34.8)
Other neurological (normal CSF)	247	57.1 ± 20.0	114 (46.2)	133 (53.8)
Epilepsy	113	58.1 ± 19.7	62 (54.9)	51 (45.1)
Stroke	120	64.8 ± 15.4	66 (55.0)	54 (45.0)
Facial paralysis	58	52.7 ± 16.6	35 (60.3)	23 (39.7)
Polyneuropathy	138	62.8 ± 13.6	102 (73.9)	36 (26.1)
Unclear cases ( normal CSF )	92	54.4 ± 18.3	36 (39.1)	56 (60.9)
Unclear cases ( abnormal CSF )	97	49.6 ± 17.1	53 (54.6)	44 (45.4)
Control	415	48.3 ± 17.6	187 (45.1)	228 (54.9)

Our findings corroborate and extend recent observations [18] demonstrating compartment-specific ionic regulation in a limited cohort of 42 individuals (28 healthy controls and 14 patients with medically unexplained neurological symptoms). Quantitative ion-selective electrode measurements revealed substantially elevated CSF concentrations of Mg and Cl relative to serum, with correspondingly reduced Ca and K levels. Our ICP-MS analysis supports this biofluid-specific elemental regulation, demonstrating consistently significant CSF–serum concentration differences (*P* < 0.001, 2-sided Wilcoxon rank-sum test) for Mg, Cl, Ca, and K in both our control cohort (data not shown) and the complete study population across all neurological conditions examined (Fig. 1A). Under physiological conditions, CSF maintains a tightly regulated ionic composition that is actively controlled and distinct from serum, rather than being merely an ultrafiltrate of plasma [8,19]. Essential elements and biologically relevant trace elements are typically maintained within narrow concentration ranges through dedicated homeostatic and chemical equilibrium mechanisms. In contrast, nonessential trace elements often lack specific regulatory pathways. However, due to chemical similarities with essential ions, they can be passively incorporated into existing equilibria [20]. Consequently, even subtle perturbations in ion transport or exchange may disproportionately affect these unregulated elements, while homeostatic buffering dampens fluctuations in essential ions.

**Fig. 2.**
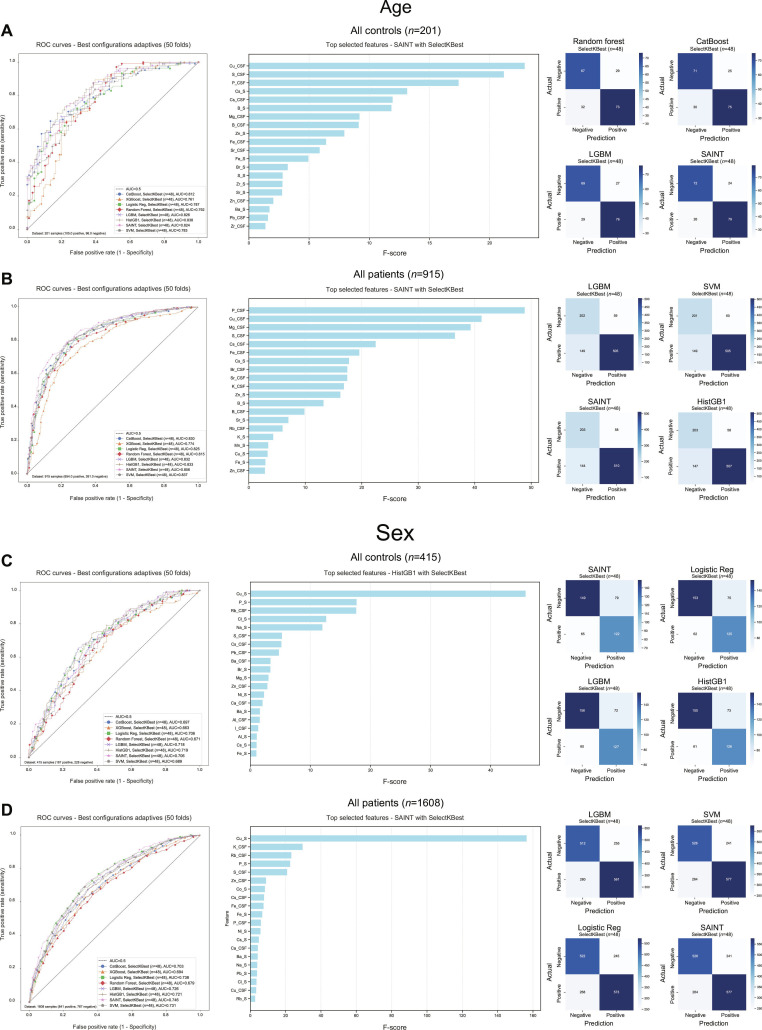
Comparative machine learning performance for age and sex prediction using element concentrations in serum and CSF. (A and B) Age prediction performance. (A) Performance comparison for age classification (youngest group: ≤ 34 years versus oldest group: >60 years) in neurologically asymptomatic controls (*N* = 201). HistGB1 ensemble classifier achieved the highest AUC (0.838), followed by light gradient boosting machine (LGBM) with an AUC of 0.826, SAINT with an AUC of 0.824, and CatBoost with an AUC of 0.812. Copper in CSF (Cu_CSF) was the most discriminative feature, followed by sulfur in CSF (S_CSF). (B) Performance in all patients (*N* =915). SAINT ensemble classifier showed superior performance (AUC = 0.856), with P in CSF (P_CSF) and Cu in CSF (Cu_CSF) as top-ranking features. (C and D) Sex prediction performance. (C) Performance comparison for sex classification in neurologically asymptomatic controls (*N* = 415). HistGB achieved the highest AUC (0.719), followed by LGBM (0.718). Cu in serum (Cu_S) was the most important feature, followed by P in serum (P_S). (D) Performance in all patients (*N* = 1,608). SAINT ensemble classifier was the best performer (AUC = 0.746), with Cu in serum (Cu_S) as the top discriminative feature.

Demographic analysis revealed that aging is characterized by systematic and compartment-specific shifts in element concentrations, with particularly pronounced significant increases in CSF levels of P, S, Fe, Cu, Zn, and Pb that were independent of corresponding serum trends (Fig. 1B). Sex-associated differences were restricted to increased CSF concentrations of Rb and Cs in females compared to males (Fig. 1C). To quantitatively assess the discriminative power of these age-related elemental changes, we employed machine learning algorithms to classify individuals according to biologically defined age strata, comparing the youngest group (≤34 years) against the oldest group (>60 years) of the population based on their elemental profiles. Age cutoffs of 34 and 60 years were defined according to Lehallier et al. [21], who identified these as proteomic inflection points across the human lifespan. Age prediction demonstrated robust performance across multiple algorithms, with the histogram gradient boosting (HistGB) ensemble classifier achieving the highest area under the curve (AUC) of 0.838 in controls and self-attention and intersample attention transformer (SAINT) achieving 0.856 in the full patient cohort (Fig. 2A and B). In contrast, sex classification showed slightly lower performance, with HistGB achieving AUCs of 0.719 in controls and SAINT reaching 0.746 in all patients, representing the maximum performance observed (Fig. 2C and D). The superior classification accuracy for age compared to sex confirms that element concentration profiles exhibit stronger age-related than sex-related patterns, providing quantitative validation of our demographic findings.

To test whether BBB and blood–CSF barrier dysfunction could explain this biofluid elemental dysregulation, we evaluated the albumin quotient, the ratio of albumin concentration in CSF to albumin concentration in serum, a well-established index of barrier permeability [22]. Albumin is produced exclusively in the liver and cannot be synthesized within the CNS. Therefore, its presence in CSF directly indicates BBB compromise, establishing the albumin quotient as a reliable marker of barrier integrity [22]. Correlation analysis (Fig. 1B) revealed that the albumin quotient exhibited significant age-dependent changes that paralleled those observed in CSF elements (B, Mg, S, K, Fe, Cu, Zn, Sr, and Cs). Among these elements, 5 (Mg, S, Fe, Cu, and Zn) demonstrated dysregulation specifically in the CSF compartment rather than in serum. These findings support the notion that barrier alteration contributes to CNS elemental dysregulation.

Notably, electrolyte (including Na and Ca) concentrations remained unaffected by BBB damage indices, despite our observation that these electrolytes exhibited significantly higher serum concentrations compared to CSF under physiological conditions (data not shown). Given this pronounced concentration gradient, age-related BBB deterioration would be expected to result in passive equilibration and subsequent elevation of these electrolytes in CSF. However, the absence of such changes demonstrates that independent active regulatory mechanisms exist to maintain ion-specific concentration gradients between serum and CSF, effectively counteracting the effects of increased barrier permeability. This selective vulnerability pattern suggests a fundamental mechanistic distinction. The observed age-related changes are predominantly linked to protein-bound elements rather than free ionic species, as protein-associated elements exhibit greater susceptibility to passive diffusion across compromised barriers.

Age-related declines in nutrient transporter function may account for diminished metal clearance, as proteomic profiling of human brain microvessels has identified significant alterations in transporter expression during aging [23]. Key BBB transporters including the glucose transporter GLUT1 and lipoprotein receptor LRP1 decline in older individuals [16], potentially limiting brain clearance of metal-binding proteins. This interpretation is consistent with dynamic contrast imaging studies revealing subtle BBB leakage in hippocampal regions of cognitively normal older adults [24], supporting the finding that albumin quotient increases with physiological aging. Alternatively, this pattern may reflect impaired clearance of protein–metal complexes due to altered CSF outflow dynamics [25].

While aging is known to compromise barrier integrity through pericyte loss, endothelial dysfunction, and altered tight junction expression, our data provide direct evidence of these physiological transitions through CSF-based elemental alteration with unprecedented population-scale resolution. The molecular perturbations underlying barrier dysfunction coincide with elevated CNS levels of oxidative stress mediators and metal-dependent protein aggregates, features characteristic of age-related neurodegeneration, indicating that elemental dysregulation could represent both a consequence of and contributor to aging-associated neurological decline [26]. These observations prompted us to further focus on patients with neurological disorders, including age-related diseases such as Alzheimer’s disease, Parkinson’s disease, and amyotrophic lateral sclerosis, where disrupted metal homeostasis and barrier dysfunction are established pathological features [16].

### Neurological diseases induce distinctive compartment-specific elemental perturbations

**Fig. 3.**
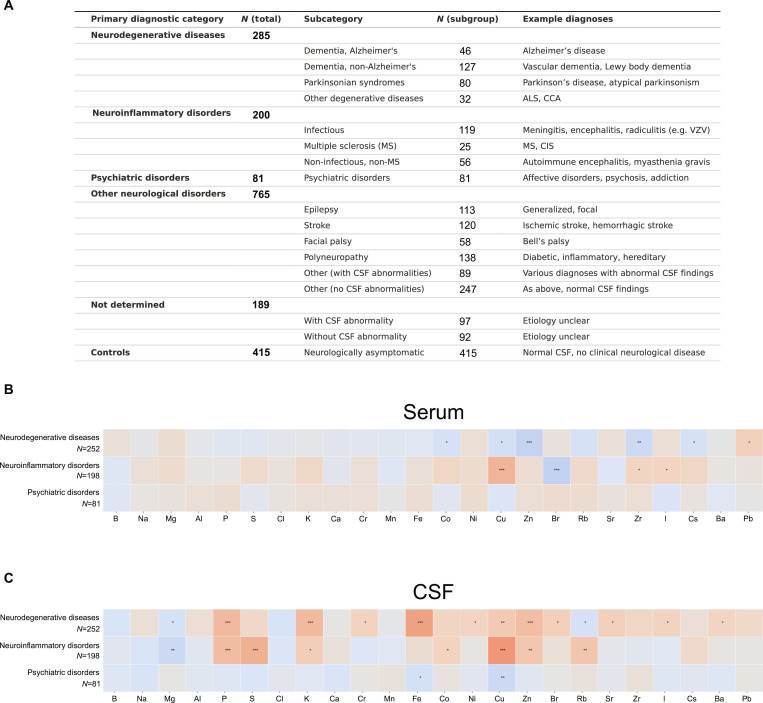
Compartment-specific elemental alterations across major neurological disease categories. (A) Overview of diagnostic categories in the neurologically affected cohort (*N* = 1,193; 904 with a single pathology, 251 with 2 concurrent conditions, and 38 with 3 comorbidities) and neurologically asymptomatic controls (*N* = 415). (B and C) Heatmaps illustrating disease-associated element concentration changes in serum (B) and CSF (C) relative to controls (*N* = 415). Color scale represents direction and magnitude of change (red, increased; blue, decreased), with statistical significance indicated by asterisks (*P < 0.05, **P < 0.01, ***P < 0.001; 2-sided Wilcoxon rank-sum test). CSF demonstrated greater sensitivity to pathological changes than serum (23 versus 10 significant alterations).

Having established a normative baseline for age-related elemental changes, we next investigated how specific neurological diseases alter these elemental profiles. To systematically characterize disease-specific elemental perturbations, patients were categorized into 3 primary diagnostic classifications with corresponding subcategories. These comprised neurodegenerative disorders (including Alzheimer’s disease, non-Alzheimer’s dementias, Parkinsonian syndromes, and other degenerative pathologies), neuroinflammatory conditions [subdivided into infectious etiologies, multiple sclerosis (MS) spectrum disorders, and non-MS autoimmune diseases], psychiatric

disorders, and additional neurological conditions including epilepsy, cerebrovascular disease, and cranial neuropathies. Reflecting the complexity of real-world clinical practice, analysis revealed that among the patients, 904 presented with a single pathology, while 251 had 2 concurrent conditions and 38 had 3 comorbidities (Fig. 3A).

Comparative analysis of the 3 primary diagnostic classifications revealed distinct compartment-specific disease signatures, with CSF exhibiting markedly superior sensitivity to pathological perturbations compared to serum (23 versus 10 statistically significant elemental alterations, respectively; Fig. 3B and C).

In the systemic circulation, neurodegenerative disorders were characterized by consistent depletion of Co, Cu, Zn, Zr, and Cs relative to neurologically normal controls. Conversely, neuroinflammatory diseases demonstrated contrasting elevations in Cu, Zr, and I concentrations above baseline levels.

CSF elemental dysregulation proved more extensive and diagnostically discriminative. Neurodegenerative pathologies were distinguished by significant accumulation of P, K, Cr, Fe, Ni, Cu, Zn, Br, Sr, I, and Ba compared to asymptomatic controls. Neuroinflammatory disorders induced selective enrichment of P, S, K, Co, Cu, Zn, and Rb beyond control thresholds. Psychiatric conditions exhibited minimal perturbations across both biological compartments, with elemental profiles remaining largely indistinguishable from control populations.

Building on the distinct CSF and serum elemental signatures observed across the 3 primary diagnostic classifications, we next conducted a detailed analysis of elemental content across all 14 diagnostic subgroups to further elucidate disease-specific patterns and their diagnostic implications.

### CSF and serum element signatures for neurological disease stratification

Analysis of patient cohorts demonstrated sex-specific differences in baseline serum trace element concentrations, with female controls exhibiting significantly elevated levels of Na, P, Cl, Co, Cu, and Br compared to male controls (Fig. 1C). In contrast, CSF trace element profiles showed minimal sex-related variation (Fig. 1C), indicating that peripheral blood differences do not translate to the CNS compartment. Given the negligible sex differences observed in CSF, we prioritized maximizing cohort size to enhance statistical power rather than enforcing strict sex matching across diagnostic groups. To control for potential sex-related confounding effects, we systematically documented male to female ratios for each diagnostic category.

This demographic analysis revealed heterogeneous sex distributions among diagnostic classifications. Ten diagnostic groups demonstrated male predominance, one group (psychiatric disorders) exhibited balanced sex representation, while 3 groups, inflammatory conditions/MS spectrum disorders (including clinically isolated syndrome), other neurological disorders without CSF abnormalities, and epilepsy, showed female predominance.

**Fig. 4.**
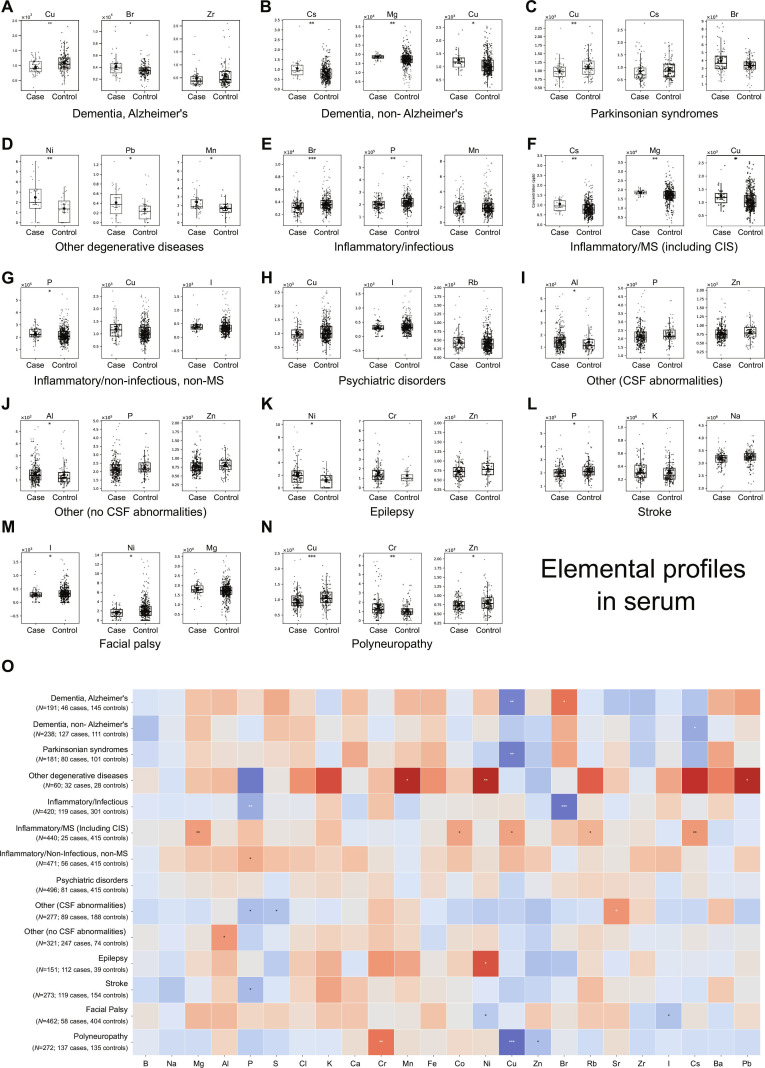
Serum element signatures across neurological diagnostic subgroups. (A to N) Box plots showing serum concentrations of the 3 most altered elements for each diagnostic subgroup versus neurologically asymptomatic controls: Alzheimer’s disease (A), non-Alzheimer’s dementias (B), Parkinsonian syndromes (C), other neurodegenerative diseases (D), neuroinflammation (E), MS (F), non-infectious/non-MS inflammatory disorders (G), psychiatric disorders (H), other neurological conditions with (I) or without (J) CSF abnormalities, epilepsy (K), stroke (L), facial palsy (M), and polyneuropathy (N). (O) Heatmap summarizing serum elemental alterations across all diagnostic subgroups. Color intensity reflects direction and magnitude of change (red, increased; blue, decreased). Statistical significance indicated by asterisks (*P < 0.05, **P < 0.01, ***P < 0.001; 2-sided Wilcoxon rank-sum test). Despite modest individual changes, distinct and reproducible element signatures emerged across diagnostic categories.

This analysis of serum element content across all 14 diagnostic subgroups (Fig. 4) revealed that several elemental alterations initially appeared potentially explained by cohort sex composition. Cu, P, and Br concentrations declined in male-skewed groups, while Cu and Co increased in the female-predominant MS cohort, mirroring baseline sex differences observed in controls. However, numerous elemental perturbations persisted despite sex ratio imbalances, establishing their independence from demographic confounders. The most compelling evidence emerged from male-dominated cohorts exhibiting opposing trends. Alzheimer’s disease patients showed prominent Br elevation, directly contradicting the expected decline based on sex composition alone. This divergent pattern implicates pathology-specific mechanisms rather than sex bias.

Disease-intrinsic element signatures emerged across multiple diagnostic categories, demonstrating pathology-specific alterations that were independent of sex distribution patterns and transcending demographic profiles. The category other degenerative disease consistently exhibited elevated Mn, Ni, and Pb irrespective of sex composition. Inflammatory conditions demonstrated distinct disease-specific patterns. MS showed increases in Mg, Rb, and Cs, while non-MS inflammatory diseases exhibited P elevation regardless of male-to-female ratios. Epilepsy patients displayed Ni elevation, whereas facial palsy showed reductions in both Ni and I independent of demographic composition. Polyneuropathy exhibited a unique pattern with elevated Cr alongside reduced Zn that persisted across sex distributions.

**Fig. 5.**
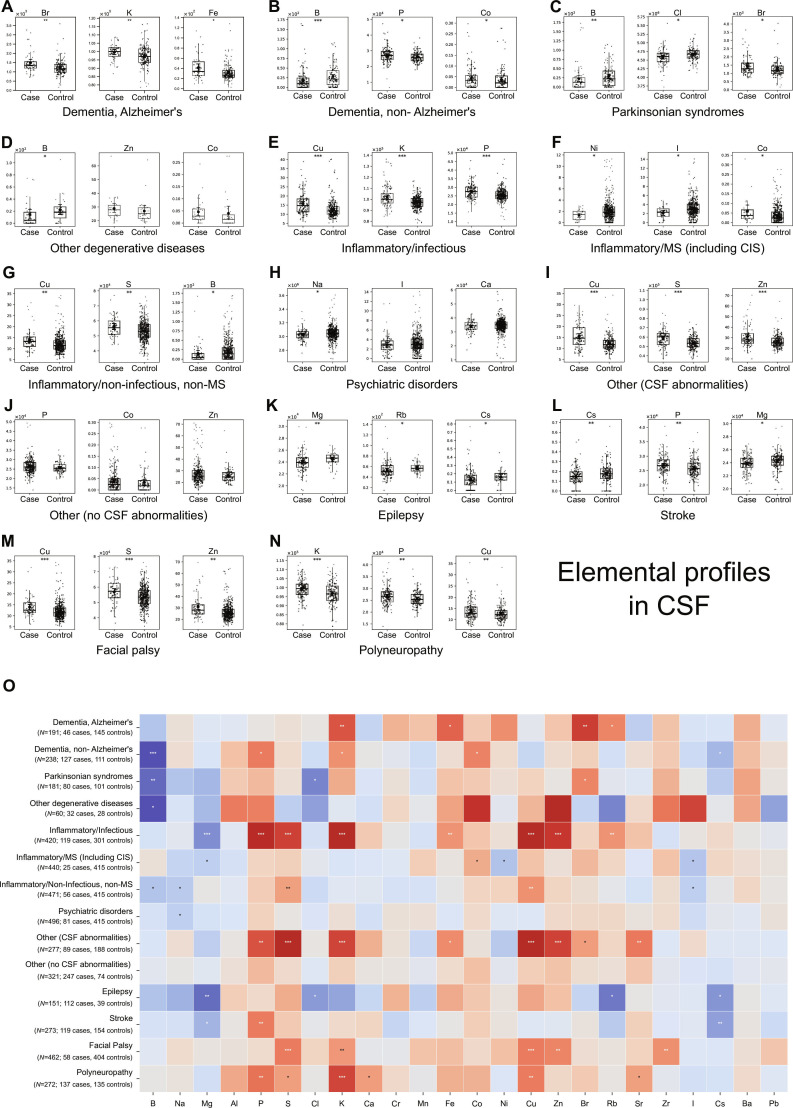
Disease-specific CSF elemental signatures across neurological diagnostic subgroups. (A to N) Box plots displaying CSF concentrations of the 3 most altered elements for each diagnostic subgroup versus controls, using the same subgroup organization as Fig. 4. (O) Heatmap summarizing CSF elemental alterations across diagnostic subgroups relative to controls. Color scale represents direction and magnitude of change (red, increased; blue, decreased) compared to the control group, with statistical significance levels indicated by asterisks (*P < 0.05, **P < 0.01, ***P < 0.001; 2-sided Wilcoxon rank-sum test). CSF elemental profiles revealed substantial and diagnostically informative alterations, with each condition exhibiting characteristic elemental perturbations that distinguished it from controls and other diagnostic categories.

Anticipating that CSF would exhibit greater pathological discrimination than serum due to its direct reflection of CNS pathophysiology, we performed comprehensive analysis of the 14 diagnostic subgroups, revealing distinct and mechanistically relevant element signatures (Fig. 5). Our comprehensive elemental profiling of neurological disorders reveals a fundamental pathophysiological dichotomy between conditions driven by BBB compromise and those exhibiting disease-intrinsic elemental dysregulation. Inflammatory and infectious conditions, in conjunction with the other CSF abnormalities group, demonstrated a characteristic BBB disruption signature, manifested by Mg depletion accompanied by selective elevation of S, Fe, Cu, and Zn, consistent with passive transvascular leakage as the predominant pathogenic mechanism.

Conversely, neurodegenerative disorders, inflammatory MS, and epileptic syndromes exhibited distinct elemental perturbation patterns independently of BBB compromise. Non-Alzheimer’s dementias and Parkinsonian disorders demonstrated pronounced B depletion, a phenotype previously associated with decreased brain functions and cognitive performance [27], while Br accumulation in Alzheimer’s disease and Parkinsonian syndromes corroborated previous postmortem investigations demonstrating that elevated cerebral Br concentrations correlate with advanced amyloid and tau neuropathology [28].

**Fig. 6.**
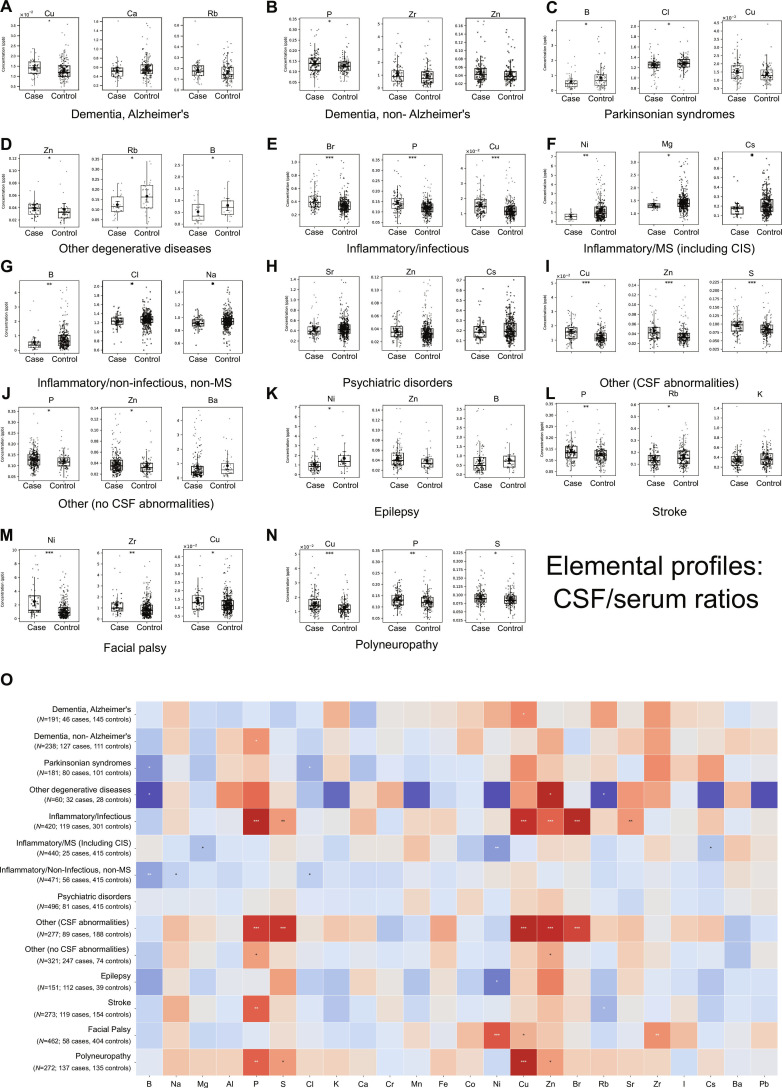
CSF-to-serum elemental ratios reveal disease-specific transport and barrier dysfunction patterns. (A to N) Box plots depicting CSF-to-serum ratios for the 3 elements showing the most significant differences between cases and controls across diagnostic subgroups, organized as in Figs. 4 and 5. (O) Heatmap summarizing CSF-to-serum ratio alterations across 24 elements and 14 diagnostic subgroups. Colors indicate direction and magnitude of ratio changes (red, increased; blue, decreased) relative to controls. Statistical significance denoted by asterisks (*P < 0.05, **P < 0.01, ***P < 0.001; 2-sided Wilcoxon rank-sum test). CSF-to-serum ratios provided enhanced sensitivity for detecting compartmental elemental redistribution, blood–brain barrier dysfunction, and disease-specific transport mechanisms, revealing both conserved and divergent patterns across neurological conditions.

Our findings reveal disease-specific element signatures that reflect distinct underlying pathogenic mechanisms rather than generalized BBB dysfunction. Cs depletion emerged as a potential hallmark of acute neuronal hyperexcitability in stroke and epilepsy, while K and Rb flux imbalances characterized Alzheimer’s dementia in the absence of accompanying BBB major disruption. While Cs has been used experimentally to modulate neuronal excitability [29], its role as a biomarker for excitotoxicity assessment remains unexplored. These mechanistic insights may warrant further exploration, for example, whether B supplementation could be beneficial in neurodegenerative conditions (as previously suggested by others [27,30]), the potential for Cs monitoring to serve as a tool for excitotoxicity assessment, and the possibility that Br evaluation could emerge as a biomarker for neurodegenerative progression. To quantify disease-related disruptions in barrier function, we calculated CSF-to-serum ratios for all measured elements across diagnostic subgroups (Fig. 6). CSF-to-serum ratios substantially eliminated sex-related biases observed in individual compartment measurements, isolating CNS-intrinsic signatures. For instance, while MS patients exhibited elevated serum Cu levels, CSF Cu concentrations remained unchanged relative to controls, resulting in CSF-to-serum Cu ratios that showed no significant difference between groups, demonstrating successful normalization of sex-related confounders in our age-matched cohort.

Quantitative CSF-to-serum ratio assessment confirmed passive leakage patterns characteristic of barrier dysfunction. In inflammatory and infectious conditions, elevated CSF concentrations of S, Cu, Zn, and Sr directly correlated with increased CSF-to-serum ratios for 4 elements, validating compromised barrier selectivity and potential passive transbarrier flux from systemic circulation. Conversely, Parkinsonian disorders and the group including other neurodegenerative disease demonstrated maintained serum B homeostasis alongside significant CSF B depletion, yielding markedly reduced CSF-to-serum B ratios. The preservation of normal systemic B equilibrium concurrent with selective CNS depletion, as demonstrated by reduced ratios, suggests CNS-specific transport dysfunction operating independently of peripheral factors such as dietary intake or renal clearance.

Critically, each neurological subgroup exhibited a distinctive and reproducible CSF-to-serum ratio signature, substantiating that CSF-to-serum ratio analysis, analogous to CSF measurements alone, elucidates disease-specific patterns that establish elemental profiles as promising biomarker candidates for patient stratification, as CSF-to-serum ratios provide enhanced sensitivity for detecting compartmental elemental redistribution, BBB dysfunction, and disorder-specific transport mechanisms, offering diagnostic information complementary to established protein-based biomarkers.

### Synergistic elemental and biological markers enhance neurological disease classification

**Fig. 7.**
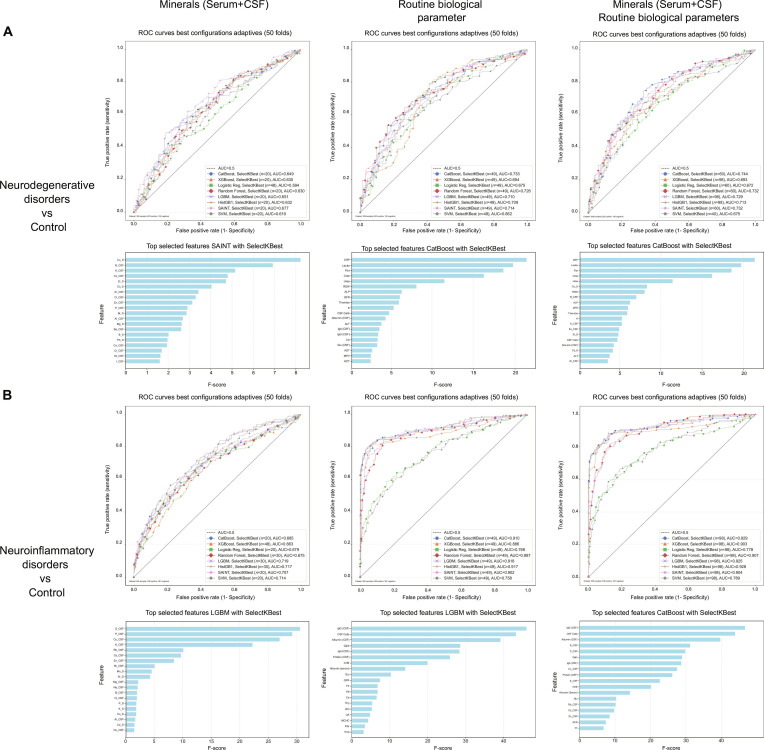
Machine learning classification performance for neurological disorders using elemental and routine biological parameters. (A) Classification of neurodegenerative disorders versus controls. ROC curves demonstrate performance of 7 machine learning algorithms across 3 feature sets: element concentrations from serum and CSF (left panel), routine serum and CSF biological parameters (center panel), and combined elemental-routine features (right panel). SAINT ensemble classifier showed optimal performance using elements alone (AUC = 0.677), while CatBoost demonstrated superior discrimination with routine parameters (AUC = 0.733). The integrated approach combining element concentrations and routine biological parameters yielded maximal classification accuracy with CatBoost (AUC = 0.744). Feature importance analyses beneath each ROC panel identify critical discriminative biomarkers, with Cu in serum (Cu_S) exhibiting highest importance among elemental features, while CRP parameters emerged as dominant contributors within routine biological measurements. (B) Classification of neuroinflammatory disorders versus controls. Machine learning performance using element concentrations (left panel) showed LGBM achieving best results (AUC = 0.719), with CSF S and P identified as most important features. Routine biological parameters (center panel) demonstrated good performance, with LGBM reaching AUC = 0.918. Combined elemental and routine parameters (right panel) achieved improved classification, with CatBoost attaining AUC = 0.929. Feature importance analysis revealed synergistic contributions from both CSF elemental S and P and routine parameters including IgG concentration in the CSF and the cell number in the CSF.

To evaluate the diagnostic utility of these mechanistically distinct elemental perturbation patterns, we systematically investigated whether elemental biomarkers provide orthogonal diagnostic information beyond routine clinical parameters or reflect overlapping pathophysiological processes. Machine learning classification analyses revealed that elemental biomarkers exhibit consistent diagnostic potential across primary diagnostic categories. Integration of elements with routine biological parameters significantly enhanced performance (Fig. 7). Across both neurodegenerative disorders and neuroinflammatory conditions, adding elemental biomarkers to routine parameters led to higher maximum AUC values compared to using routine parameters alone, demonstrating the diagnostic value of supplementation with elemental biomarkers. For neurodegenerative disorders, the combined approach (CatBoost AUC = 0.744) systematically outperformed routine parameters alone (CatBoost AUC = 0.733), while for neuroinflammatory conditions, the enhancement was even more pronounced, improving from excellent standalone performance [light gradient boosting machine (LGBM) AUC = 0.918] to near-optimal discrimination (CatBoost AUC = 0.929).

This universal enhancement across disparate neurological conditions establishes a fundamental principle. Element signatures and routine biological parameters capture complementary rather than redundant pathophysiological information. The consistent superiority of integrated approaches validates a paradigm shift toward biomarker augmentation rather than replacement, where elemental profiles provide orthogonal diagnostic information that synergizes with established clinical measures. The complementary relationship reflects the unique capacity of elemental biomarkers to detect condition-specific ionic dysregulation and BBB dysfunction, pathophysiological processes that remain largely inaccessible through conventional protein-based or inflammatory biomarkers.

Notably, our classification approach, while demonstrating robust diagnostic potential, represents a simplified methodology relative to the inherent complexity of neurological disorders, which encompass multiple subtypes, severity grades, and pathological variants.

The observed performance suggests that larger datasets enabling stratification into more homogeneous disease subgroups could reveal substantially enhanced discriminatory power, as refined element signatures may capture subtle pathophysiological patterns specific to individual disease phenotypes that remain obscured in broader categorical classifications.

These findings could have important implications for personalized medicine in neurology, where integrating elemental and routine biomarkers may support earlier detection and more precise diagnosis and potentially inform therapeutic monitoring strategies.

Recent preprints by Xu et al. [31] and An et al. [32] have advanced similar analytical frameworks for extracting disease-relevant signals from proteomic data, underscoring the growing utility of omics-driven pipelines in health stratification. Xu et al. developed several unsupervised methods to characterize proteome changes during the awakening response in saliva samples from healthy individuals and those with mitochondrial disease (MitoD). These methods included delta clustering for co-regulation networks, elasticity profiling for nonlinear dynamics, and tensor decomposition for multidimensional representation. Similarly, An et al. introduced ProtAIDe-Dx, a deep joint-learning model trained on plasma proteomics from over 17,000 participants, to provide probabilistic diagnoses across neurodegenerative conditions such as Alzheimer’s disease, Parkinson’s disease, and frontotemporal dementia. These studies align with our approach in utilizing high-throughput omics data and machine learning to capture systemic differences between health and disease.

However, key distinctions arise from the omics modality: While proteomics quantifies thousands of proteins reflecting downstream functional states (e.g., enzyme activity and signaling pathways), metallomics, as employed here, targets elemental signatures (e.g., trace metals like Zn, Fe, and Cu) that serve as upstream cofactors and regulators in biological processes. Metallomics complements proteomics by revealing metal dyshomeostasis linked to oxidative stress, protein misfolding, and neurodegeneration, phenomena often overlapping with proteomic alterations in MitoD [31] and Alzheimer’s/Parkinson’s disease [32]. Our pipeline, applied to CSF and serum metallomic profiles, uses ensemble machine learning (e.g., random forests and gradient boosting) for feature extraction and classification, achieving robust separation of health states with fewer dimensions (20 to 50 elements) compared to the high-dimensional proteomic spaces (>3,000 proteins) in Xu et al. and An et al. To comprehensively evaluate both linear and nonlinear relationships within these profiles, we benchmarked a diverse suite of supervised learning architectures using rigorous stratified repeated cross-validation and optimized regularization, ensuring that performance variability reflects the distinct sensitivity of mathematical frameworks to biological signals rather than the capture of technical noise. This lower dimensionality may enhance generalizability and reduce overfitting risks in smaller cohorts, although it limits granularity on protein-specific pathways. Future integrative analyses combining metallomic and proteomic datasets could yield hybrid models with superior predictive power, bridging elemental regulation to protein dynamics for more comprehensive disease insights. Similarly, extending such integration to neuroimaging-based brain age prediction models, which reveal how cumulative clinical risk factors like hypertension accelerate structural brain aging in population cohorts [33], or to retinal fundus imaging prompted by longitudinal clinical metadata for accurate brain volume estimation [34], could create multimodal frameworks that combine molecular elemental signatures with accessible structural proxies, enhancing early detection and stratification across neurological disorders.

The integration of element signatures with established biomarkers may help shape a hierarchical diagnostic framework, where initial cost-effective screening could identify at-risk populations for targeted evaluation with specialized tests. If validated, such an approach may provide broad clinical utility. Moreover, the accessibility of elemental measurements could make them suitable for longitudinal monitoring of treatment response and disease progression.

## Conclusion

In conclusion, our comprehensive analysis of paired serum and CSF elemental profiles across 1,608 individuals establishes the largest systematically standardized database of neurological patients, processed using uniform analytical protocols, enabling direct cross-disease comparison. We identify compartment-specific signatures that enable robust disease stratification through mechanistically distinct pathways. BBB-mediated passive diffusion correlates with inflammatory disorders versus disease-intrinsic metal dysregulation characterizing neurodegenerative conditions. These reproducible, disease-associated perturbations remain independent of systemic variations and demonstrate complementary classification performance when integrated with routine clinical parameters through ensemble learning approaches.

The findings advance our understanding of metal biology in neurological disease while demonstrating artificial intelligence (AI)-driven metallomics for precision disease stratification. Machine learning algorithms revealed subtle elemental perturbations that enhance patient subtyping beyond conventional approaches, with biofluid-specific signatures providing mechanistic insights into CNS pathophysiology.

Elemental profiling may offer particular value in neurological conditions lacking established biomarkers, exemplified by Parkinson’s disease, where no reliable CSF or serum biomarker has yet achieved clinical implementation [35]. In such conditions, element signatures could help address diagnostic gaps while providing complementary biochemical information, potentially enhancing multimodal biomarker panel performance when integrated with protein-based measurements.

Future prospective studies will be essential to systematically validate these findings against comprehensive lifestyle and pharmacological datasets across diverse populations, establishing clinical utility while addressing potential ethnic and geographic variations in baseline profiles. Ultimately, this cost-effective approach may establish a foundation for hierarchical diagnostic strategies, where accessible first-line screening could help direct patients toward appropriate specialized testing, potentially improving diagnostic accuracy and healthcare resource allocation.

## Methods

### Ethical approval

This study was approved by the Ethics Committee of the Saarland Medical Board (Reference 08/11). Clinical data were collected as part of routine diagnostics and pseudonymized for subsequent analysis. In accordance with German law and the ethics board approval, oral informed consent was obtained from all patients, as written consent was not required for this type of retrospective analysis of routine diagnostic samples.

### Reagents and materials

All reagents used were of analytical grade. Ultrapure water (Milli-Q, 18.2 MΩ·cm) and Suprapur grade nitric acid [69% (v/v), Roth] were employed for sample dilution and standard preparation. Certified metal-free tubes (VWR) were used for the preparation of both samples and standards. A semiquantitative calibration standard was prepared by diluting a multi-element stock solution (reference 85006.186, VWR), containing 100 mg/l of Al, Ag, As, B, Ba, Be, Bi, Ca, Cd, Co, Cr, Cu, Fe, K, Li, Mg, Mn, Mo, Na, Ni, Pb, Sb, Se, Sr, Ti, Tl, V, and Zn, in 5% HNO₃ (v/v). An ICP-MS tuning solution (Agilent Technologies), containing 1 μg/l of Ce, Co, Li, Tl, and Y in 2% HNO₃ (v/v), was used to optimize signal intensity and instrument stability. Additionally, an in-house biological fluid reference was used as a control to ensure long-term reproducibility, with acceptable variation set below 15%.

### Sample collection and preparation

Serum and CSF samples were obtained from the biobank of the Department of Neurology at the University Hospital Saarland. These specimens constitute residual material from routine clinical diagnostics and were originally collected as part of standard diagnostic procedures, independent of the present study. For serum preparation, venous blood was drawn into serum-separating tubes and allowed to clot at room temperature for 30 min, followed by centrifugation at 3,000*g* for 10 min at room temperature. CSF samples were collected via lumbar puncture performed under sterile conditions and were subsequently centrifuged at 400*g* for 10 min at room temperature to remove cellular components. The resulting supernatants were aliquoted into sterile, 1.5-ml polypropylene microcentrifuge tubes and immediately frozen. All aliquots were stored at –20 °C in temperature-monitored, access-controlled freezers. Samples were pseudonymized and managed using a laboratory information management system, which also recorded associated metadata including patient age, sex, diagnosis, and date of sampling. Detailed demographic and clinical characteristics of the study cohort, including group-specific age and sex distributions for the control group and all 14 neurological categories, are summarized in Table 1. All procedures conformed to institutional standard operating procedures for the handling and long-term storage of human biological specimens, ensuring biospecimen integrity, regulatory compliance, and full traceability.

Serum and CSF samples were diluted at a 1:25 ratio using 1% (v/v) HNO₃. Nitric acid was selected due to its compatibility with ICP-MS instrumentation and its ability to effectively solubilize elements present in the samples. The dilution factor was optimized to reduce matrix effects and ensure accurate quantification of trace elements. This simple dilution step provides a fast, straightforward, and reproducible sample preparation method suitable for analysis.

### ICP-MS analysis

The element composition of biological fluids was determined using multi-elemental analysis, following established protocols adapted for biological matrices. Diluted samples were analyzed using an Agilent 7850 single quadrupole ICP-MS system, featuring an integrated SPS 4 autosampler for automated sample introduction and micromist nebulizer. The collision cell was set to helium mode for all elements. The operating conditions included radio frequency power of 1,550 W, sample depth of 7 mm, plasma argon gas flow rate of 15 l/min, auxiliary argon gas flow rate of 0.9 l/min, nebulizer argon gas flow rate of 1.05 l/min, stabilization time of 30 s, collision helium gas flow rate of 5 ml/min, and integration time per isotope of 600 ms.

An 8-point calibration (7 concentration levels) was applied to the following elements, using a custom-made standard: ^47^Ti, ^51^V, ^75^As, ^111^Cd, and ^238^U. A semiquantitative analysis was applied to the following elements identified as relevant for neurological biomarker analysis: ^11^B, ^23^Na, ^24^Mg, ^27^Al, ^28^Si, ^31^P, ^34^S, ^35^Cl, ^39^K, ^43^Ca, ^45^Sc, ^52^Cr, ^55^Mn, ^56^Fe, ^59^Co, ^60^Ni, ^63^Cu, ^66^Zn, ^79^Br, ^85^Rb, ^88^Sr, ^89^Y, ^90^Zr, ^93^Nb, ^115^In, ^118^Sn, ^127^I, ^133^Cs, ^137^Ba, ^139^La, ^140^Ce, ^141^Pr, ^146^Nd, ^147^Sm, ^182^W, ^205^Tl, and ^208^Pb.

The SQ approach is based on a 2-point semicalibration using a 28-elemental standard and a 1% HNO₃ solution as a blank. The following elements were used for semiquantification, each at a concentration of 20 μg/l: Al, Ag, As, B, Ba, Be, Bi, Cd, Co, Cr, Cu, Fe, Li, Mg, Mn, Mo, Ni, Pb, Sb, Se, Sr, Ti, Tl, V, and Zn. The concentrations of the remaining elements were interpolated based on those included in the calibration, using response factors that consider their isotopic mass, isotopic abundance, and ionization energy. The element composition serves as the basis for defining the Element Bio Profile, with measured concentrations providing the input data for machine learning algorithms. For quality control purposes, elements with more than 50% of measurements below the limit of detection (LOD) were excluded from further analysis to ensure data reliability. For the remaining elements, concentrations below the LOD were replaced with zero values during the imputation process, as these represent genuinely absent or negligible concentrations in the biological matrix.

### Study population and data processing

The dataset analyzed consists of clinical samples from patients presenting various neurological, psychiatric, or inflammatory disorders, along with neurologically asymptomatic control subjects (*N* = 415). The total study population comprised 1,608 subjects distributed across 6 primary diagnostic categories: neurodegenerative diseases (*N* = 285), neuroinflammatory disorders (*N* = 200), psychiatric disorders (*N* = 81), other neurological disorders (*N* = 765), undetermined (*N* = 189), and controls (*N* = 415).

Patients were classified into distinct diagnostic categories, each further subdivided into specific subcategories based on clinical diagnosis. Neurodegenerative diseases included dementia, Alzheimer’s (*N* = 46), dementia, non-Alzheimer’s (*N* = 127), Parkinsonian syndromes (*N* = 80), and other degenerative diseases (*N* = 32, including amyotrophic lateral sclerosis). Neuroinflammatory disorders comprised infectious conditions (*N* = 119, including meningitis, encephalitis, and radiculitis), MS (*N* = 25, including MS and clinically isolated syndrome), and non-infectious, non-MS conditions (*N* = 56, including autoimmune encephalitis and myasthenia gravis). Psychiatric disorders (*N* = 81) included affective disorders, psychosis, and addiction. Other neurological disorders were subdivided into epilepsy (*N* = 113), stroke (*N* = 120), facial palsy (*N* = 58), polyneuropathy (*N* = 138), other with CSF abnormalities (*N* = 89), and other with no CSF abnormalities (*N* = 247). Undetermined cases were classified as either with CSF abnormality (*N* = 97) or without CSF abnormality (*N* = 92), both with unclear etiology. Controls were neurologically asymptomatic individuals with normal CSF and no clinical neurological disease (*N* = 415), serving as the reference group. The cohort comprised 904 patients with single pathologies, 251 patients with 2 concurrent conditions, and 38 patients with 3 comorbidities, reflecting the complex clinical reality of neurological practice where multimorbidity is common. Category sample sizes reflect inclusive counting, where comorbid patients are assigned to all relevant categories, resulting in total assignments (1,520) exceeding unique patients (1,193). Each analysis systematically compared a specific pathological group against matched controls, ensuring comparable age distributions.

Broad-category AUC modeling was performed to assess the robustness of metallomic signatures under realistic clinical heterogeneity and complements the granular 14-subgroup analysis. Within-category pooling tests whether shared pathophysiological mechanisms generate discriminable elemental patterns, which is clinically relevant for initial diagnostic stratification.

The study population comprised 1,608 individuals with an overall mean age of 54.5 ± 18.4 years. This included 851 males (mean age 55.9 ± 18.2 years) and 757 females (mean age 52.9 ± 18.5 years).

All patients were diagnosed by 2 experienced neurologists according to the standardized diagnostic criteria established by the German Society for Neurology [Deutsche Gesellschaft für Neurologie (DGN)] and, where applicable, the German Society for Psychiatry and Psychotherapy, Psychosomatics and Neurology. This approach ensured consistent classification across all diagnostic groups, including heterogeneous categories such as “other neurodegenerative diseases”, for which specific DGN guidelines were applied. For MS, we applied the revised 2017 McDonald criteria as incorporated in the DGN guideline on MS diagnosis and therapy. This standardized diagnostic framework minimizes heterogeneity within disease categories and enhances the interpretability of disease-specific patterns.

### Routine clinical parameters

Routine biological parameters were systematically collected from the central laboratory of the University Hospital of Saarland. The dataset comprised 41 conventional biomarkers from serum spanning multiple physiological systems: electrolyte balance (Na, K, Ca), renal function (creatinine, estimated glomerular filtration rate, urea), and metabolic status (glucose, uric acid). Hepatic function (total protein, albumin, aspartate aminotransferase, alanine aminotransferase, γ-glutamyl transferase, alkaline phosphatase, bilirubin, cholinesterase), pancreatic enzymes (amylase, pancreatic amylase, lipase), cardiac/muscle injury markers (creatine kinase, lactate dehydrogenase, troponin T), hematological parameters (leukocytes, erythrocytes, hemoglobin, hematocrit, mean corpuscular volume, mean corpuscular hemoglobin, mean corpuscular hemoglobin concentration, red cell distribution width, thrombocytes, mean platelet volume), coagulation studies (prothrombin time, international normalized ratio, partial thromboplastin time, thrombin time, fibrinogen), inflammatory markers (C-reactive protein), and immunological assessments (immunoglobulins IgG, IgA, IgM). In parallel, CSF analysis included cell count, glucose concentration, albumin levels, immunoglobulins (IgG, IgA), and proteins. The CSF/serum albumin quotient was calculated using paired CSF and serum albumin measurements to evaluate BBB integrity.

All laboratory measurements adhered to German quality assurance standards.

### Age matching procedure

Age distribution comparisons between patients and control subsets were performed using the Mann–Whitney *U* test (2-sided, *P* value threshold = 0.05). An iterative bidirectional algorithm selected the largest control subset maintaining nonsignificant age differences (*P* > 0.05) while maximizing statistical power. The algorithm was operated by systematically removing subjects with extreme ages from both patient and control groups when age ranges differed between groups. When mean ages differed, samples from the 5th or 95th percentiles were iteratively removed from the group with more extreme values. The process continued for up to 20 optimization cycles, with each cycle recalculating the Mann-Whitney *U* test *P* value and retaining the subset configuration with the highest *P* value. For cases where no subset achieved *P* > 0.05, the algorithm selected the configuration yielding the highest available *P* value. A minimum group size of 5 subjects was required for both patients and controls. Age matching success was validated through final Mann–Whitney *U* testing before proceeding to biomarker analysis.

### Statistical analysis

The statistical analysis assessed the differences in the concentrations of 48 biomarkers (elements or other biological markers) between matched patient groups and controls using a standardized heatmap visualization approach. For each biomarker–pathology pair, available data were extracted and missing values were systematically excluded to create complete case datasets for statistical analysis. Statistical significance of group differences was assessed using different approaches based on variable type. For binary pathological variables (coded as 0/1 like pathologies or sex), Spearman correlation coefficients were calculated between biomarker concentrations and pathological status, while *P* values were determined using the Mann–Whitney *U* test (2-sided alternative) comparing biomarker distributions between affected and unaffected groups. For continuous pathological variables, both correlation coefficients and *P* values were derived from Spearman rank correlation analysis.

Results were visualized using heatmaps generated with seaborn library (Seaborn.pydata.org), where each cell represents an elemental–pathology pair with colors corresponding to the strength of observed correlations (colormap: coolwarm, centered at 0, range: −0.3 to +0.3). Statistical significance was visually annotated using asterisks overlaid on correlation values: *P < 0.05, **P < 0.01, and ***P < 0.001.

For the 3 most statistically significant elemental–pathology associations identified through heatmap analysis, complementary boxplot visualizations were generated to illustrate the distribution patterns and effect sizes. Boxplots were created for both serum and CSF matrices across all pathological categories studied. Each boxplot displays the distribution of element concentrations between cases and age-matched controls, with individual data points overlaid as strip plots and mean values indicated by point estimates with confidence intervals. Statistical significance markers (asterisks) were added above boxplots for associations with *P* < 0.05. Outliers were filtered using a 5× interquartile range criterion to improve visualization clarity while maintaining data integrity. The boxplots were sorted by ascending *P* values to prioritize the most significant associations, providing a comprehensive view of biomarker concentration differences across neurological, psychiatric, and inflammatory disorders in both biological matrices.

### Machine learning binary classification analysis

Variable selection was performed using the SelectKBest method from the scikit-learn library. This step systematically selects variables most predictive of the pathological state based on a univariate criterion [analysis of variance (ANOVA) *F* test: f_classif]. Three distinct sets of explanatory variables were analyzed separately: the 48 element concentrations (MPPC set), routine biological parameters (BLOOD set), and a combination of elements and routine parameters (TOT set). Multiple configurations of selected variables were evaluated to identify the optimal subset size delivering the best predictive performance. For the 48 element concentrations, *k* values of 10, 22, 30, and 48 were tested; for routine biological parameters, *k* values of 10, 22, 30, and 41 were evaluated; and for the combined set, *k* values of 10, 22, 30, 41, 70, and 89 were assessed. All features were standardized using StandardScaler (mean-centered and scaled to unit variance), ensuring comparability across biomarkers.

Multiple supervised classification algorithms were implemented with regularized parameters optimized to reduce overfitting. Logistic regression employed L2 regularization with C = 0.08, solver = “liblinear”, class_weight = “balanced”, and max_iter = 400. Support vector machine used a radial basis function kernel with default parameters (C = 1.0, gamma = “scale”, class_weight = “balanced”). Random forest utilized an ensemble of decision trees with n_estimators = 20, max_depth = 2, max_features = “sqrt”, and class_weight = “balanced”. XGBoost classifier implemented gradient boosting with enhanced regularization (n_estimators = 100, learning_rate = 0.005, max_depth = 3, min_child_weight = 5, gamma = 1.0, scale_pos_weight = 1, eval_metric = “auc”). CatBoost classifier employed gradient boosting with categorical feature handling and automatic class weight balancing (iterations = 50, depth = 2, learning_rate = 0.05, auto_class_weights = “Balanced”, eval_metric = “MCC”). LightGBM classifier used a gradient boosting framework with regularization (n_estimators = 100, max_depth = 2, learning_rate = 0.05, min_child_samples = 30, reg_alpha = 0.1, class_weight = “balanced”). HistGB classifier implemented scikit-learn’s gradient boosting with L2 regularization (max_iter = 120, max_depth = 2, learning_rate = 0.04, l2_regularization = 0.05, class_weight = “balanced”). The SAINT ensemble classifier employed a multi-head attention-inspired ensemble implemented using VotingClassifier with soft voting strategy, combining 5 diverse base estimators: SVM with RBF kernel (C = 0.7), SVM with polynomial kernel (C = 1.0, degree = 3), random forest with square root feature selection (n_estimators = 100, max_depth = 8, max_features = “sqrt”), random forest with log_2_ feature selection (n_estimators = 100, max_depth = 8, max_features = “log2”), and extra trees classifier (n_estimators = 150, max_depth = 8, max_features = “sqrt”). All base estimators employed balanced class weighting.

While comparative statistical analyses were performed independently for each biofluid to characterize compartment-specific signatures, multivariate machine learning models utilized an integrated 48-variable feature set (comprising 24 elements from CSF and 24 from serum) to leverage the orthogonal diagnostic information provided by central and systemic compartments and evaluate their synergistic predictive value.

### Cross-validation procedure and data leakage prevention

Model performance was assessed using a stratified *K*-fold cross-validation strategy with 50-fold numbers. All preprocessing steps, including imputation, normalization, and feature selection, were performed independently within each cross-validation fold to prevent data leakage. Specifically, for each fold, the imputation parameters, scaling factors, and feature selection criteria were fitted exclusively on the training subset and then applied to the corresponding test subset. The complete analysis pipeline was implemented using scikit-learn Pipeline objects ensuring that no information from test sets influenced training procedures, all transformations were consistently applied across folds, and feature selection was performed independently for each training fold. This approach guarantees that model performance estimates reflect true generalization capability rather than overfitted performance due to data leakage.

A methodological consideration in this study is the reliance on internal cross-validation without prospective external validation in an independent cohort. To mitigate potential overfitting and ensure robust performance estimation, our analytical framework incorporates multiple stringent safeguards: stratified 50-fold cross-validation to preserve class balance across folds, pipeline-enclosed preprocessing to eliminate data leakage, and explicit regularization constraints (L2 penalties in logistic regression; gamma and min_child_weight hyperparameters in XGBoost) to prevent model overparameterization.

### Evaluation metrics

Classification performance was quantified using receiver operating characteristic (ROC) curve and AUC for evaluating overall discriminative power, sensitivity (true positive rate) and specificity (true negative rate), and confusion matrix providing detailed counts of true positive (TP), false positive (FP), true negative (TN), and false negative (FN) outcomes. Feature importance was assessed either directly from the classifier (e.g., random forest and CatBoost) or via scores from SelectKBest (for linear models), identifying biomarkers contributing significantly to predictive power.

### Software implementation

All analyses were conducted using Python (version 3.8) with a comprehensive suite of machine learning and data analysis libraries. The core machine learning framework was built using scikit-learn, which provided the primary algorithms for logistic regression, support vector machines, random forest, and HistGB, along with essential tools for data preprocessing (SimpleImputer, StandardScaler), feature selection (SelectKBest), and cross-validation (StratifiedKFold). Advanced gradient boosting implementations were incorporated through specialized libraries including XGBoost for XGBClassifier, CatBoost for automated categorical feature handling and class balancing, and LightGBM for efficient gradient boosting with regularization. The SAINT ensemble classifier was implemented using scikit-learn’s VotingClassifier, combining multiple base estimators with soft voting strategies. Data handling and numerical computations were performed using pandas for structured data handling and NumPy for array operations, while statistical tests and correlations were computed using Scipy particularly for Mann–Whitney *U* tests and Spearman correlation coefficients. Visualization components, including heatmaps for exploratory analysis, ROC curves for model evaluation, confusion matrices, and feature importance plots, were generated using matplotlib as the base plotting framework and seaborn for enhanced statistical visualizations and correlation matrices.

## Data Availability

Data are available upon reasonable request.
